# Synthesis, Biomacromolecular Interactions, Photodynamic NO Releasing and Cellular Imaging of Two [RuCl(qn)(Lbpy)(NO)]X Complexes

**DOI:** 10.3390/molecules26092545

**Published:** 2021-04-27

**Authors:** Luna Song, Hehe Bai, Chenyang Liu, Wenjun Gong, Ai Wang, Li Wang, Yi Zhao, Xuan Zhao, Hongfei Wang

**Affiliations:** 1 Key Laboratory of Chemical Biology and Molecular Engineering of the Education Ministry, Institute of Molecular Science, Shanxi University, Taiyuan 030006, China; sxdxsln@163.com (L.S.); 18435202616@163.com (H.B.); cy18834854183@163.com (C.L.); sxdxgwj@163.com (W.G.); aiwang@sxu.edu.cn (A.W.); 2 Institute of Environmental Science, College of Chemistry and Chemical Engineering, Shanxi University, Taiyuan 030006, China; wangli@sxu.edu.cn; 3 Shanxi Key Laboratory of Pharmaceutical Biotechnology, Taiyuan 030006, China; zhaoyisws@163.com; 4 Department of Chemistry, University of Memphis, Memphis, TN 38152, USA; xzhao1@memphis.edu

**Keywords:** ruthenium complex, nitric oxide, photodynamic, spectra, biomacromolecules

## Abstract

Two light-activated NO donors [RuCl(qn)(Lbpy)(NO)]X with 8-hydroxyquinoline (qn) and 2,2′-bipyridine derivatives (Lbpy) as co-ligands were synthesized (Lbpy_1_ = 4,4′-dicarboxyl-2,2′-dipyridine, X = Cl^−^ and Lbpy_2_ = 4,4′-dimethoxycarbonyl-2,2′-dipyridine, X = NO_3_^−^), and characterized using ultraviolet–visible (UV-vis) spectroscopy, Fourier transform infrared (FT-IR) spectroscopy, nuclear magnetic resonance (^1^H NMR), elemental analysis and electrospray ionization mass spectrometry (ESI-MS) spectra. The [RuCl(qn)(Lbpy_2_)(NO)]NO_3_ complex was crystallized and exhibited distorted octahedral geometry, in which the Ru–N(O) bond length was 1.752(6) Å and the Ru–N–O angle was 177.6(6)°. Time-resolved FT-IR and electron paramagnetic resonance (EPR) spectra were used to confirm the photoactivated NO release of the complexes. The binding constant (K_b_) of two complexes with human serum albumin (HSA) and DNA were quantitatively evaluated using fluorescence spectroscopy, Ru-Lbpy_1_ (K_b_~10^6^ with HSA and ~10^4^ with DNA) had higher affinity than Ru-Lbpy_2_. The interactions between the complexes and HSA were investigated using matrix assisted laser desorption ionization-time of flight mass spectrometry (MALDI-TOF-MS) and EPR spectra. HSA can be used as a carrier to facilitate the release of NO from the complexes upon photoirradiation. The confocal imaging of photo-induced NO release in living cells was successfully observed with a fluorescent NO probe. Moreover, the photocleavage of pBR322 DNA for the complexes and the effect of different Lbpy substituted groups in the complexes on their reactivity were analyzed.

## 1. Introduction

During the past 30 years, the function of nitric oxide (NO) in physiological and pathological roles was extensively investigated. NO has been regarded as an important gasotransmitter signaling molecule in regulating blood pressure, the nervous system, immune responses, cellular apoptosis and fighting viral infections [1,2,3,4,5]. The overall effect of NO depends on the concentration of NO [6,7]. At low nanomolar concentration, NO provides a pro-survival effect, while at relatively high µM level, it will arrest the cellular respiration and cause apoptosis in cancers on account of the inhibition of mitochondrial cytochrome c oxidase (CcO) or activated several pro-apoptotic caspase signaling pathways [8,9,10,11]. Studies show that NO is also involved in post-translational modifications and nitrosylation plays a vital role in regulating the function of biomolecules and disease treatment [12,13].

Utilizing of exogenous NO donors becomes a strategy to deliver NO to a physiological target and regulate it to release quantitatively. However, the application of many NO-donating agents has limitations because of lacking the localized accumulation of NO to ensure apoptosis in a targeted site. Nitrosylruthenium complexes are promising NO donors because the released reactive radical NO upon exposure to light can be delivered to physiological targets for photodynamic therapy. Moreover, nitrosylruthenium complexes could also serve as drugs with the lower toxic side effects, modest photosensitivity and inherent stability under physiological environments [14,15,16]. Therefore, a great effort was taken for the synthesis and design of different types of nitrosylruthenium complexes in recent decade [17,18,19,20,21,22].

Bidentate chelators of 8-hydroxyquinoline and polypyridyl derivatives can bind metal ions due to the nitrogen and oxygen atoms in the structure. Many metal complexes with quinoline or its derivatives have shown antibacterial and antineoplastic activity. The studies showed that Ru complexes having quinoline derivatives exhibited increased antitumor activity as compared with ligands [23,24]. Identically, Ru complexes with polypyridyl derivatives have been demonstrated to be useful in binding to DNA, cellular imaging, anti-angiogenesis and anti-tumor because of beneficial chemical and photophysical properties [25,26].

**Scheme 1:**
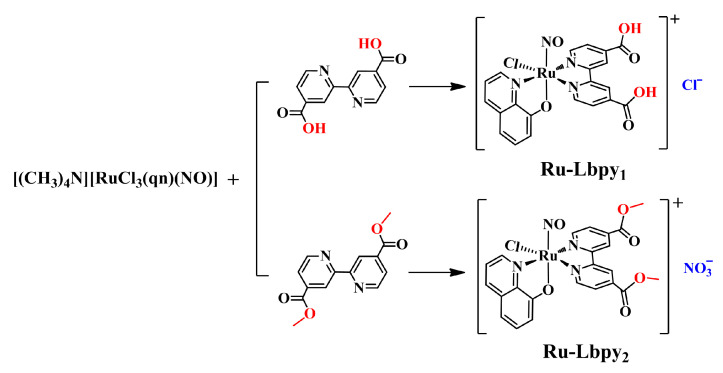
Synthesis of Ru-Lbpy_1_ and Ru-Lbpy_2_ complexes.

Different functional groups in the ligands of complexes affect the metabolism and behavior of cross cellular members, leading to the change of pharmacokinetic profile and cellular toxicity [27,28,29]. Two new nitrosylruthenium complexes [RuCl(qn)(Lbpy)(NO)]X were synthesized based on 8-hydroxyquinoline and 2,2′-bipyridine ligands (Ru-Lbpy_1_: [RuCl(qn)(4,4′-dicarboxyl-2,2′-dipyridine)(NO)]Cl and Ru-Lbpy_2_: [RuCl(qn)(4,4′-dimetho -xycarbonyl-2,2′-dipyridine)(NO)]NO_3_). Scheme 1 shows the synthetic procedure of Ru-Lbpy_1_ and Ru-Lbpy_2_ complexes.

The chemical structures were characterized using ultraviolet–visible (UV-vis) spectroscopy, Fourier transform infrared (FT-IR) spectroscopy, nuclear magnetic resonance (^1^H NMR), elemental analysis and electrospray ionization mass spectrometry (ESI-MS). NO releases from the complexes and the solvent molecule takes the vacancy as indicated in Equation: Ru-(NO) + hν + solv → Ru^III^-(solv) + NO. Furthermore, the interactions between the complexes and human serum albumin (HSA) were analyzed using fluorescence spectroscopy, matrix assisted laser desorption ionization-time of flight mass spectrometry (MALDI-TOF-MS) and electron paramagnetic resonance (EPR) spectra. The results indicated that they could bind to HSA and have a significant impact on the release of NO. The photoinduced cleavage of pBR322 DNA was also observed when the NO release from complexes occurred in the process. The photo induced NO release was further confirmed using NO selective fluorescent probe in human cervical cancer cells (HeLa cells). This work presents NO donors for the potential application of nitrosylruthenium complexes in biomedicine and photodynamic therapy.

## 2. Results and Discussion

### 2.1. Synthesis and Characterization

Two nitrosylruthenium complexes [RuCl(qn)(Lbpy)(NO)]X were synthesized by the reaction between [(CH_3_)_4_N][RuCl_3_(qn)(NO)] and dipyridine derivative ligands, and the synthesis steps are shown in Scheme 1. The two complexes were characterized by different spectroscopy techniques including ^1^H-NMR, ESI-MS, elemental analysis, UV-vis and FT-IR, as described in the experimental section and shown in supplementary information Figures S1–S9. All ^1^H chemical shift and number could be matched with these complexes to deduce the mode of these ligands coordinated to the Ru, except for the active hydrogen on the carboxyl group in Ru-bpy_1_. The ^1^H NMR spectrum of Ru-bpy_2_ (Figure S3) indicates that aromatic resonance shifts to higher field relative to Ru-bpy_1_ (Figure S1) as the modification of the methyl group. The ESI-MS spectra of complexes in methanol solution show signals at *m*/*z* 552.9485 (negative spectrum, Figure S4) and 582.9965 (positive spectrum, Figure S5) for the positively charged species [RuCl(qn)(Lbpy_1_)(NO)]Cl and [RuCl(qn)(Lbpy_2_)(NO)]NO_3_, respectively. The UV-vis spectra of complexes in dimethyl sulfoxide (DMSO) and aqueous solution show an apparent characteristic peak of metal complexes compared to ligand and the absorption of Ru-bpy_2_ (Figure S7) is red-shifted a few nanometers because of the push electron effect of the methyl group compared to Ru-bpy_1_ (Figure S6). They are stable in phosphate (PB) buffer solution (pH 7.5) and there is no change even after a week. The FT-IR spectra of Ru-bpy_1_ (Figure S8) and Ru-bpy_2_ (Figure S9) complexes show the presence of NO and Lbpy ligands, as evidenced by NO stretching frequencies (ν_NO_) at 1888 cm^−1^ in addition to their Lbpy carbonyl stretching frequencies (ν_CO_) at 1619 and 1729 cm^−1^, respectively. The lower carbonyl stretching frequency of Ru-bpy_1_ may be due to the association of carboxyl groups between the molecules.

**Figure 1:**
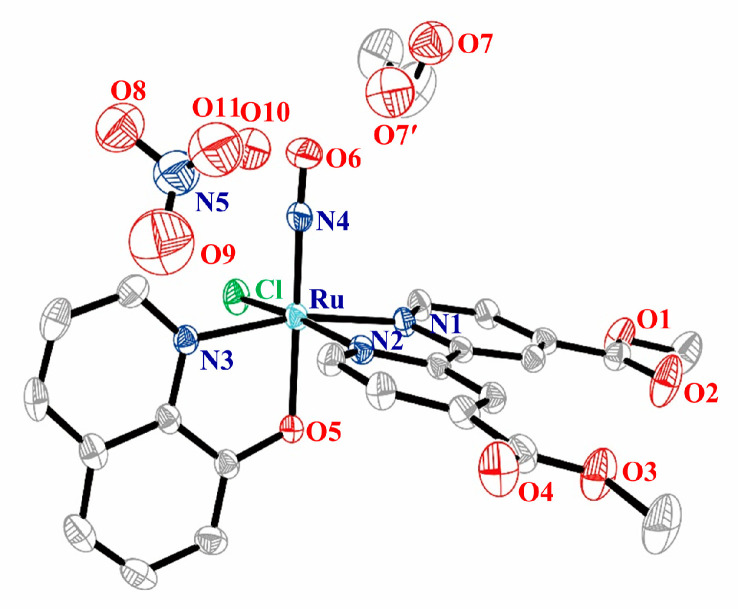
Ortep diagram of Ru-Lbpy_2_, showing 30% thermal ellipsoid probability. All hydrogen atoms in the unit cell of the complex are omitted for clarity.

The crystal of Ru-Lbpy_2_ was obtained by the slow evaporation of ethanol solvent and the structure was determined by the X-ray diffraction method. The ortep diagram of Ru-Lbpy_2_ is presented in Figure 1.

There are one positive ion [RuCl(qn)(Lbpy)(NO)]^+^, one nitrate as the counter ion and one ethanol solvent molecule in the asymmetric unit. The Ru atom is six-coordinated by the binding of the N-atom of NO, two N-atoms of the Lbpy_2_ ligand, one N-atom and one O-atom of the qn ligand, Cl^−^. Obviously, the O atom of qn ligand presents trans to NO. The nitrate ion is partially disordered, in which O8 and O9 are both 100% occupied contributing an oxygen atom each while O10 and O11 together contribute an oxygen atom with the occupancies of 40% and 60%, respectively. Moreover, the O atom of ethanol molecule is also shared by O7′ and O7 together about 39% and 61% occupied, respectively. Understandably, the thermal vibration parameters of the atoms are affected because of the measurement at room temperature. In the structure, this test alerts for possibly missed hydrogen bonds in O6-O10 and N4-O10 as indicated by short distances, however, N4, O6 and O10 atoms all have no hydrogen atom so they cannot form hydrogen bonds.

The crystallographic data and structure refinement of Ru-Lbpy_2_ are given in Table 1. The final residual factors *R* [F^2^ > 2σ(F^2^)], wR_2_ (F^2^) and goodness-of-fit (F^2^) GOOF value are 0.0663, 0.1663 and 1.023 for Ru-Lbpy_2_, respectively. In addition, selected bond lengths and angles are listed in Table 2, the Ru-N(O) bond length is 1.752 (6) and the Ru-N-O bond angle is nearly linear at 177.6 (6)°, similar to that observed in the corresponding Ru complexes [30]. The X-ray crystallographic data including atomic coordinates and equivalent isotropic temperature factors for the complex have been deposited in the Cambridge Crystallographic Data Centre (CCDC) under accession number 2054814. Atomic coordinates and equivalent isotropic temperature factors for the complex are shown in Table S1, and anisotropic displacement parameters are shown in Table S2.

### 2.2. Photo-Induced NO Release

**Figure 2:**
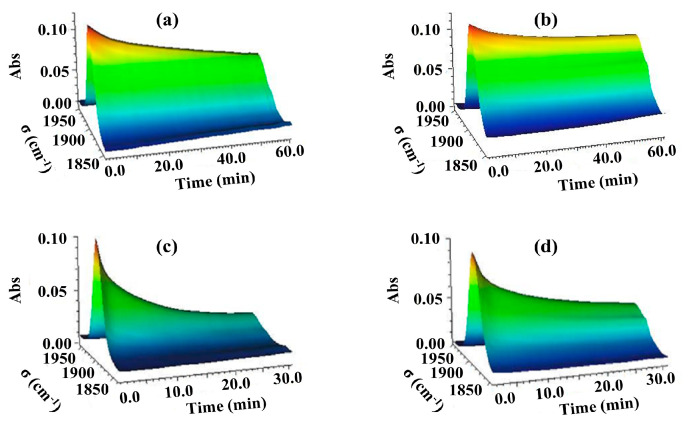
Time resolved infrared spectra of the Ru-Lbpy_1_ and Ru-Lbpy_2_ complexes upon irradiation with 420 nm light at an intensity of 75 mW∙cm^−2^ for 60 min (**a**,**b**) and Xe light at an intensity of 300 mW∙cm^−2^ (**c**,**d**) for 30 min, respectively.

In order to investigate the NO releasing capacity of [RuCl(qn)(Lbpy)(NO)]X complexes, time-resolved IR and EPR experiment were carried out. Time-resolved IR spectroscopy is an effective method to record the kinetic process of NO release. The time-resolved FT-IR spectra of two complexes upon photo irradiation are shown in Figure 2. NO vibrational frequencies of [RuCl(qn)(Lbpy)(NO)]X complexes with the Lbpy_1_ and Lbpy_2_ ligands are 1858 and 1853 cm^−1^, respectively. The released NO amount of two complexes increases along with the extended photo irradiation time. The released NO rate of Ru-Lbpy_1_ is thermodynamically faster than that of Ru-Lbpy_2_ whether it is upon a 420 nm light source or a xenon lamp. Moreover, more NO is released by xenon lamp with higher power (300 mW∙cm^−2^) than that by 420 nm illumination (75 mW∙cm^−2^) for the same complex in the same time period. The reported NO quantum yield for Ru complexes with terpyridine and bipyridine derivatives as ligands [31] and nitrosylruthenium complexes with different configurations using terpyridine derivatives as ligands is quite different [32]. It is obvious that the NO quantum yield is affected by different ligands and is related with molecular structure. The estimated NO quantum yield values (Φ_420 nm_) for Ru-Lbpy_1_ and Ru-Lbpy_2_ complexes in DMSO are 0.007 ± 0.001 and 0.005 ± 0.001, respectively. Therefore, it is possible to control the NO release from nitrosylruthenium complex by designing the ligands of the complex and adjusting location, time, and power of lamp [33,34]. 

**Figure 3:**
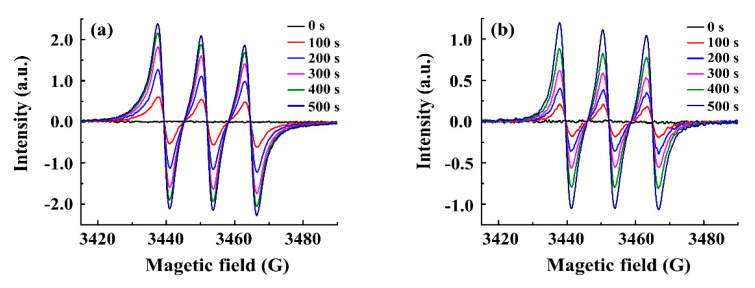
Electron paramagnetic resonance (EPR) spectra of the trapped NO from Ru-Lbpy_1_ (**a**) and Ru-Lbpy_2_ (**b**) with Hg lamp irradiation at 0 s, 100 s, 200 s, 300 s, 400 s, 500 s, respectively.

To monitor the release of NO free radical after photolysis of [RuCl(qn)(Lbpy)(NO)]-X complexes, EPR measurements were performed using trapping reagent Fe(MGD)_2_ [35]. The spectra were recorded from 0 to 500 s after the addition of Fe-MGD adduct to the complexes. There is no signal observed for the two complexes in the dark as shown by the black line in Figure 3. However, it is evident that photolyzed products display intensely hyperfine EPR triplet signals. In most cases, photolysis could produce paramagnetic Ru(III) species upon NO release as the Equation: {Ru-NO}^6^ + hν → Ru^III^-(solv) + NO according to references [36,37]. Obviously, the signal intensity of the spectra increases along with the extension of illumination time and Ru-bpy_1_ (Figure 3a) has a more significant effect on EPR signal intensity compared to Ru-bpy_2_ (Figure 3b) upon photoirradiation. The generated hyperfine splitting constant g factor by the photolysis of the complexes in 5% DMSO buffer is equal to 2.039 that is similar to the published value for NO-Fe^2+^-MGD adduct [38,39].

### 2.3. Protein Binding Properties

It is well known that the plasma contains abundant HSA, which can accumulate in tumor cells at a higher level compared to normal cells. Hence, HSA has been received more attention as being used to serve as a carrier for drug delivery in recent years, HSA-based delivery system can contribute to improving metal drugs targeted biodistribution and safety performance [40]. In order to better understand the binding mechanism between the synthesized [RuCl(qn)(Lbpy)(NO)]X complexes and HSA, the techniques such as fluorescence spectroscopy and MALDI-TOF-MS were used.

**Figure 4:**
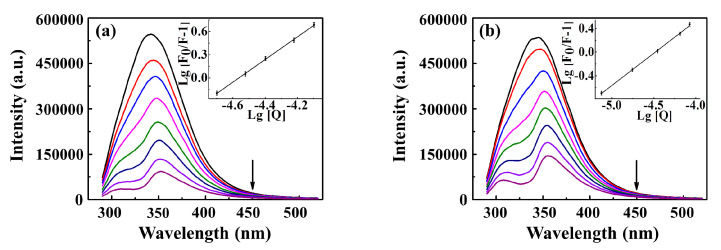
Fluorescence emission spectra of human serum albumin (HSA) (10 μM) titration with the increasing concentrations (0–80 μM) of two complexes (**a**) Ru-Lbpy_1_ and (**b**) Ru-Lbpy_2_. The excitation wavelength is 280 nm, pH 7.5, 298 K. The inset represents Stern–Volmer plot of HSA quenched by complexes, lg [F_0_/F-1] vs. lg [Q].

The endogenous fluorescence of HSA mainly stems from the chromogenic groups in the side chains of tryptophan (Trp), tyrosine (Tyr) and phenylalanine (Phe) residues. The fluorescence of HSA is quenched obviously and the wavelength is redshifted by a few nanometers with the increasing concentration of [RuCl(qn)(Lbpy)(NO)]X complexes (Figure 4). Meanwhile, the fluorescence of Tyr residue appears slightly as the concentration of the complex increases. This suggests that these complexes have a certain effect on the binding of serum protein. The binding constant (K_b_) and the binding number (n) between HSA and [RuCl(qn)(Lbpy)(NO)]X complexes can be obtained from the slope and intercept through the Scatchard formula: lg [(F_0_ − F)/F] = lg K_b_ + n lg [Q], in which Q is the complex concentration, F_0_ is the fluorescence of HSA and F is the fluorescence of HSA in the presence of the complex. The linear plots of lg [(F_0_ − F)/F] versus lg [Q] are displayed in the inner illustration. The stronger binding ability of Ru-Lbpy_1_ (4.83 ± 0.09 × 10^6^ M^−1^) than that of Ru-Lbpy_2_ (7.41 ± 0.08 × 10^4^ M^−1^) may be due to the hydrogen bonding between the carboxyl group of Ru-bpy_1_ and serum protein in addition to the static quenching (Table 3). 

**Figure 5:**
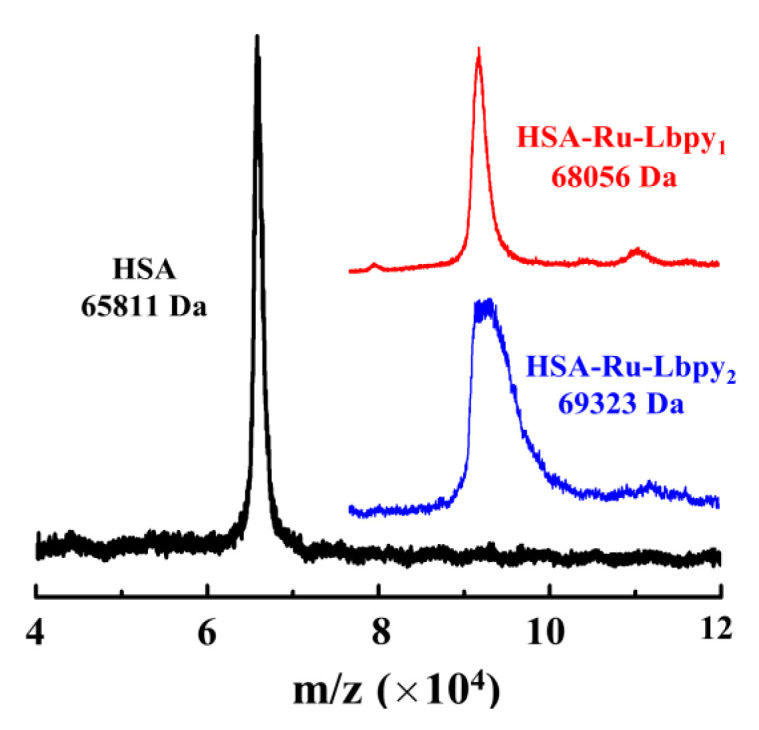
Matrix assisted laser desorption ionization-time of flight mass spectrometry (MALDI-TOF) mass spectra of HSA alone (black), Ru-Lbpy_1_-HSA complex (red) and Ru-Lbpy_2_-HSA complex (blue) in the 2,5-dihydroxybenzoic acid (DHB) matrix, respectively.

HSA molecule is a single-chain protein which contains 585 amino acid residues in a heart-like shape [41,42]. MALDI-TOF-MS spectra for native HSA, Ru-Lbpy_1_-HSA complex and Ru-Lbpy_2_-HSA complex are shown in Figure 5. The data indicate an increase in molecular weight of approximately 2245 Da for the Ru-Lbpy_1_-HSA complex and 3512 Da for the Ru-Lbpy_2_-HSA complex relative to HSA, corresponding to the molecular weight of ca. four Ru-Lbpy_1_ and five Ru-Lbpy_2_ each bound per HSA molecule, respectively. Crystal structure indicates that HSA has three domains (I–III), the data of MALDI-TOF-MS spectra suggest that 3~5 molecules are possibly encapsulated in one HSA molecule. Beside the binding site in the sub-domain, these complex molecules are possibly temporarily stored in the space between domains I, II and III of HSA. The possible binding mode for metal drugs in a HSA-based delivery system is multiple. 

**Figure 6:**
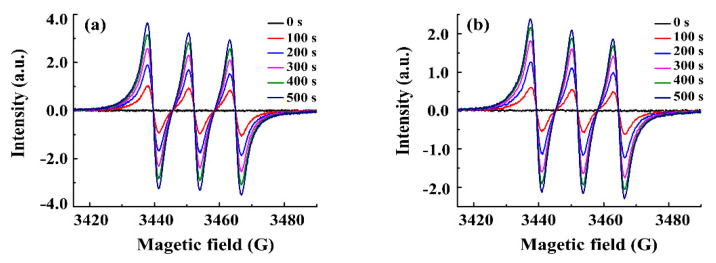
EPR spectra of the trapped NO from Ru-Lbpy_1_ (**a**) and Ru-Lbpy_2_ (**b**) in the presence of HSA with Hg lamp irradiation at 0 s, 100 s, 200 s, 300 s, 400 s, 500 s, respectively.

To observe the effect of [RuCl(qn)(Lbpy)(NO)]X complexes with HSA on the NO release, EPR technology was operated. Notably, after the interaction of Ru-Lbpy_1_/Ru-Lbpy_2_ complexes with HSA, the intensity of trapped NO under light irradiation is approximately twice more than those complexes alone in solution in EPR experiment (Figure 6). In general, HSA could be used as the carrier for the transport of these complexes in vivo to form HSA-Ru complex adduct and promote NO release upon illumination, which provides the basis for developing novel nitrosylruthenium drugs and a protein delivery system for photodynamic therapy. 

### 2.4. DNA Binding Properties

DNA is a vital biological target and the binding of metal complexes to DNA is the key to understand the mechanism of action [43]. The interactions of [RuCl(qn)(Lbpy) (NO)]X complexes and calf thymus DNA (CT-DNA) were studied by fluorescence spectroscopy and agarose gel electrophoresis. 

**Figure 7:**
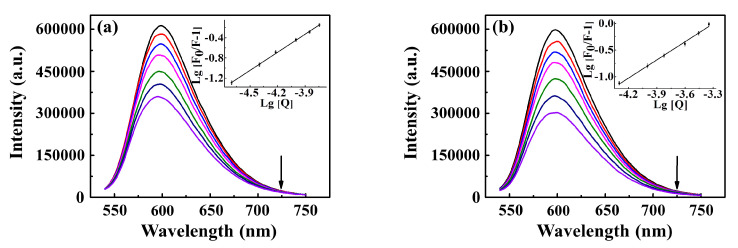
Fluorescence emission spectra of CT-DNA (170 μM) with increasing concentrations of two complexes (**a**) Ru-Lbpy_1_ and (**b**) Ru-Lbpy_2_. The excitation wavelength is 520 nm, pH 7.4, 298 K. The inset represents Stern-Volmer plot of CT-DNA quenched by complexes, lg [F_0_/F-1] vs. lg [Q].

Fluorescence spectroscopy as a sensitive technique is used to analyze the binding pattern of the CT-DNA-EB (ethidiumbromide) system with complexes. CT-DNA-EB has strong emission intensity at 598 nm because the EB aromatic ring structure is parallel inserted into the DNA double helix, avoiding fluorescence quenching from the solvent. After the addition of the Ru complexes, the fluorescence intensity decreases as the complexes replace EB in the EB-DNA system (Figure 7). The binding constant (K_b_) and the number of average binding sites (n) between them are obtained according to the Scatchard equation, Q is the DNA concentration, and F_0_ and F are the fluorescence of DNA in the absence and presence of Ru complex. The linear plots are displayed in the inner illustration. The calculated binding constant through static quenching mode is 2.14 ± 0.08 × 10^4^ and 5.52 ± 0.16 × 10^3^ M^−1^ for the Ru-Lbpy_1_ and Ru-Lbpy_2_ complexes, respectively. The average binding-site number is about one (Table 3). There is planar ring in two complexes, therefore, the binding of the Ru complexes with DNA mainly involves intercalation. Furthermore, Ru-Lbpy_1_ has stronger binding ability than Ru-Lbpy_2_, which may be related to the hydrogen bond between the carboxyl group of Ru-Lbpy_1_ and DNA.

**Figure 8:**
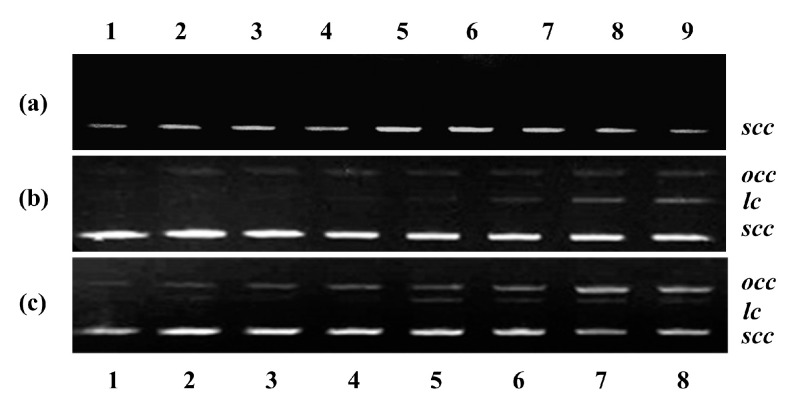
Agarose gel electrophoresis of pBR322 DNA (0.05 μg) with different concentrations of Ru-Lbpy_1_ and Ru-Lbpy_2_ complexes. (**a**) control without photoirradiation (Lane 1: DNA only; Lane 2-5: DNA + Ru-Lbpy_1_, 1, 10, 100, 200 μM; Lane 6–9: DNA + Ru-Lbpy_2_, 1, 10, 100, 200 μM) (**b**) DNA + Ru-Lbpy_1_ with photoirradiation (Lane 1: DNA only; Lane 2–8: 1, 5, 10, 25, 50, 100, 200 μM) (**c**) DNA + Ru-Lbpy_2_ with photoirradiation (Lane 1: DNA only; Lane 2–8: 1, 5, 10, 25, 50, 100, 200 μM).

In order to investigate the effect of [RuCl(qn)(Lbpy)(NO)]X complexes after illumination on plasmid pBR322 DNA configuration, agarose gel electrophoresis technology was used. It has been reported that pBR322 DNA with different configurations moves at different velocities in the electric field. The closed superhelical form (scc) for PBR322 DNA migrates the fastest while the open-circular form (occ) produces slower migration in the electrophoresis process because of the single strand breakage. In addition, if both chains of DNA are split, the linear form (lc) is produced that migrates between scc and occ [44]. The photo induced pBR322 DNA damage experiment by the complexes was carried out (Figure 8). The results show that DNA still exists in the form of the closed superhelix and the complexes (<200 μM) could not induce DNA damage in the dark (Figure 8a). However, the photocleavage of DNA takes place after interacting with the complexes by irradiation. As the concentration of Ru-Lbpy_1_ increases, DNA is cleaved into the open-loop form at first, followed by the emergence of the linear form (Figure 8b). As a comparison, DNA is mainly cut into the open-loop form for Ru-Lbpy_2_ (Figure 8c). To sum up, illumination promotes the DNA cleavage activity of the complexes and the interaction between them occurs through the light-induced reaction pathway. 

### 2.5. Cytotoxicity and Imaging of NO Releasing in Living Cell

A series of ruthenium complexes containing the different ligands against cancer cells have been reported, these complexes could be used to reduce tumor growth, and some complexes could increase mean survival time with a relatively low cytotoxicity and genotoxicity in non-tumor cells [45,46,47,48,49,50,51,52,53]. The studies on the Ru complexes with multi-mixed ligands provide a strategy to search for effective leading compound and shed light on the mechanism of action. Cytotoxicity and photocytotoxicity of the two [RuCl(qn)(Lbpy)(NO)]X complexes were evaluated against HeLa cells. Unexpectedly, the cytotoxicity of the two complexes is very weak for the tested tumor cell, as shown in Figure S10A,B. The IC_50_ is 290 μM and 320 μM for Ru-Lbpy_1_ and Ru-Lbpy_2_ complexes, respectively. Photocytotoxicity is shown in Figure S10. The photocytotoxicity for the complexes could be observed, although they are weak. The half maximal inhibitory concentration (IC_50_) is 200 μM and 215 μM for the two complexes, respectively. 

**Figure 9:**
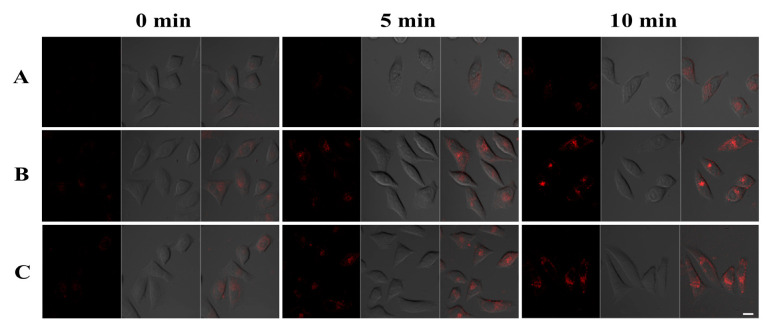
The red confocal microscopy images of HeLa cells treated with DAX-J2 (NO probe) (**A**) and DAX-J2 + Ru-Lbpy_1_ (**B**) and DAX-J2 + Ru-Lbpy_2_ (**C**) for 0, 5, 10 min by a light-emitting diode (LED) light (0.3 A, 420 nm). Excitation was performed with 561 nm laser and emission wavelength was recorded in the range of 579–701 nm. The scale bars: 20 μm.

To investigate whether the complexes could be transported into cell or NO could be released upon photoirradiation of [RuCl(qn)(Lbpy)(NO)]X complexes, fluorescence imaging assay for NO in living cells was evaluated with a confocal fluorescence microscope and DAX-J2 as NO-sensitive fluorescent probe. To reduce the interference of the probe on the cells, DAX-J2 (5 µM) was used in the subsequent biological imaging assays. HeLa cells have almost no fluorescence or very weak fluorescence due to endogenous NO when they are treated with DAX-J2 whether it is in a dark condition or upon light irradiation (Figure 9A). However, DAX-J2-loaded Hela cells are treated with synthetic nitrosylruthenium complexes and then exposed to a light-emitting diode (LED) lamp continuously, distinct red fluorescence is observed (Figure 9B,C). Thus, the exogenous NO release from [RuCl(qn)(Lbpy)(NO)]X can enter into cells and photo-induced NO release could be observed in the cellular environment. 

## 3. Materials and Methods

### 3.1. Materials

RuCl_3_·xH_2_O, 8-hydroxyquinoline (qn) and tetramethylammonium chloride ((CH_3_)_4_NCl) were purchased from aladdin (Beijing, China). 4,4′-dicarboxyl-2,2′-dipyridine (Lbpy_1_) was obtained from Henghua Technology Co. LTD (Jinan, China). FeSO_4_·7H_2_O was purchased from J&K (Beijing, China). N-methyl-D-glucaminedithiocarbamate (MGD) was purchased from Dojindo (Kumamoto, Japan). Albumin human serum (HSA), calf thymus DNA (CT-DNA), ethidiumbromide (EB) solution and pBR322 DNA were provided from Solarbio (Beijing, China). NO probe (DAX-J2) was purchased from AAT Bioquest Inc (Sunnyvale, CA, USA). DMEM medium (high glucose), fetal bovine serum (FBS), penicillin/streptomycin, 1 × PBS and tyrisin used in cell culture were obtained from Sangon Biotech (Shanghai, China). Cell counting kit-8 (CCK8) was purchased from Sigma (St. Louis, MO, USA). Human cervical cancer cells (HeLa cells) were obtained from Shanxi key laboratory of pharmaceutical biotechnology (Taiyuan, China). Other chemicals and solvents used were obtained from locally available suppliers. 

### 3.2. Measurements

^1^H NMR spectra were measured by Bruker 600 MHz nuclear magnetic resonance spectrometer. Mass spectra were obtained by Thermo Q Exactive field orbital well cyclotron resonance mass spectrometer. IR spectra (solid infrared, liquid infrared and time-resolved infrared) were carried out by Thermo Nicolet iS50 FT-IR infrared spectrometer. UV-vis spectra were measured by a Thermo Evolution-220 spectrometer. Elemental analysis was measured by a vario EL CUBE. XRD was conducted by a Bruker Smart Apex II diffractometer. EPR was measured by a Bruker EMXPLUS10/12 spectrometer. Fluorescence spectra were measured by a FluoroMax-4 spectrofluorometer. The molecular weight of protein was analyzed by Bruker Ultraflex MALDI-TOF-MS spectrometer. Agarose gel electrophoresis was performed by DYY-6C electrophoresis apparatus of Beijing Liuyi Biological Technology Co., Ltd (Beijing, China). A cytotoxicity test was measured by SpectraMax iD5 microplate reader. Confocal microscopy images were analyzed by a LSM-880 confocal laser scanning microscope.

### 3.3. Synthesis

The two complexes were synthesized from the [(CH_3_)_4_N][RuCl_3_(qn)(NO)] precursor. The [(CH_3_)_4_N][RuCl_3_(qn)(NO)] complex was synthesized according to the published literature [54].

#### 3.3.1. Synthesis of [RuCl(qn)(Lbpy_1_)(NO)]Cl (Ru-Lbpy_1_)

The solution of [(CH_3_)_4_N][RuCl_3_(qn)(NO)] (45.6 mg, 0.1 mM) in ethanol and water (10 mL + 10 mL) was added to the Lbpy_1_ ligand at 1:1 molar ratio. The reaction was stirred for 8 h at 85 °C and separated by filter to remove unreacted ligand. The filtrate was evaporated under reduced pressure and dried in vacuo. The crude product was purified by silica gel column chromatography with CH_2_Cl_2_:EtOH:H_2_O = 3:2:0.5, which was dried in vacuo to obtain the reddish-brown solid. Yield: 8.6 mg (15%). ^1^H NMR (600 MHz, DMSO) δ 9.37 (d, J = 5.6 Hz, 1H), 9.22 (d, J = 4.6 Hz, 1H), 8.89 (s, 1H), 8.84–8.76 (m, 2H), 8.24 (d, J = 5.5 Hz, 1H), 7.97 (dd, J = 8.1, 5.1 Hz, 1H), 7.76 (s, 2H), 7.53 (t, J = 7.9 Hz, 1H), 7.47 (d, J = 8.0 Hz, 1H), 6.86 (d, J = 7.7 Hz, 1H). ESI-MS: m/z 552.9485 (calcd. *m*/*z*^-^ for [M-2H^+^-Cl^−^]^−^ 552.9494). Absorption maxima (DMSO): 328, 397, 476 nm. IR (KBr pellet, cm^-1^): 3137–3697, 1888, 1619, 1552, 1502, 1470, 1373. Anal. Calcd for C_21_H_14_Cl_2_N_4_O_6_Ru: C, 42.73; H, 2.39; N, 9.49. Found: C, 42.85; H, 2.30; N, 9.38.

#### 3.3.2. Synthesis of [RuCl(qn)(Lbpy_2_)(NO)]NO_3_ (Ru-Lbpy_2_)

4,4′-Dimethoxycarbonyl-2,2′-dipyridine (Lbpy_2_) ligand was synthesized as previously described [55] with some modifications. To the solution of 4,4′-dicarboxyl-2,2′-dipyridine (312 mg, 1.2 mM) in methanol (45 mL) was added concentrated sulfuric acid (6 mL). After refluxing for 24 h, the solution was cooled and poured into 45 mL water to form a white slurry. The pH value of the slurry was adjusted to 8~9 with 1 M NaOH. The crude product was filtered, dried in vacuo and purified by silica gel column chromatography with CH_2_Cl_2_:CH_3_OH = 50:1 to obtain the white solid. Yield: 234 mg (79%). ^1^H NMR (600 MHz, CDCl_3_) δ 8.97 (s, 2H), 8.89–8.85 (m, 2H), 7.91 (dd, J = 4.9, 1.5 Hz, 2H), 4.00 (s, 6H). 

The synthesis steps of [RuCl(qn)(Lbpy_2_)(NO)]NO_3_ (Ru-Lbpy_2_) as follows: the solution of [(CH_3_)_4_N][RuCl_3_(qn)(NO)] (91.0 mg, 0.2 mM) in ethanol (20 mL) was added to the Lbpy_2_ ligand at 1:1 molar ratio. The reaction was stirred for 8 h at 85 °C and separated by filter to remove unreacted ligand. The filtrate was evaporated under reduced pressure and dried in vacuo. The crude product was purified by silica gel column chromatography with CH_2_Cl_2_:EtOH:saturated NaNO_3_ = 10:1:0.04, which was dried in vacuo to obtain the reddish-brown solid. Yield: 30 mg (23%). [RuCl(qn)(Lbpy_2_)(NO)]NO_3_∙EtOH crystals suitable for X-ray crystallography were grown by slow diffusion in ethanol solution. ^1^H NMR (600 MHz, DMSO) δ 9.66 (d, J = 5.2 Hz, 1H), 9.54 (s, 1H), 9.48 (s, 1H), 9.26 (s, 1H), 8.83 (d, J = 8.4 Hz, 1H), 8.52 (d, J = 5.1 Hz, 1H), 8.08 (d, J = 5.0 Hz, 1H), 8.05-7.98 (m, 2H), 7.57–7.49 (m, 2H), 6.85 (d, J = 7.4 Hz, 1H), 4.08 (s, 3H), 3.97 (s, 3H). ESI-MS: *m*/*z* 582.9965 (calcd *m*/*z*^+^ for [M-NO_3_^−^]^+^ 582.9958). Absorption maxima (DMSO): 331, 403, 481 nm. IR (KBr pellet, cm^−1^): 2918, 2848, 1888, 1729, 1504, 1361, 1319, 1262, 1107. Anal. Calcd for C_25_H_24_ClN_5_O_10_Ru: C, 43.45; H, 3.50; N, 10.13. Found: C, 43.57; H, 3.53; N, 10.02.

### 3.4. Photo-Induced NO Release

Time-resolved IR spectra were carried out to investigate the NO releasing capacity for [RuCl(qn)(Lbpy)(NO)]X complexes. These complexes (3.5 mg) were dissolved in deuterated DMSO and the solutions were added to the infrared sample cell consisting of two rounded CaF_2_ windows. The measured wavelength was from 1850 to 1950 cm^−1^ and the resolution was 4 cm^−1^. A Xe lamp (HSX-F300, 0.3 W∙cm^−2^) in the absence and presence of 420 nm band-pass filter was used to irradiate samples, which were kept at a specific distance from the filter to maintain the constant irradiated power. To eliminate the produced heat in the illumination process, radiating facilities such as radiator fan and air conditioning were used. The light irradiation time was 60 min in the wavelength of 420 nm filter and 30 min with Xe lamp. The quantum yield of two complexes were estimated at the wavelength of λ = 420 nm according to the equation: Φ_420 nm_ = n N_A_ h c/E λ (where n was the number of reactant molecules in mol, N_A_ was Avogadro constant, h was Plank constant, *c* was the speed of light and E was the absorbed photon energy), in which the absorbed photon energy was obtained by the formula E = I t S T (I was the power density of incident light; t was the time; S was the effective absorption area; T was the transmittance) [56].

X-band EPR spectra of [RuCl(qn)(Lbpy)(NO)]X complexes were detected continuously using the following parameters: microwave frequency, 9.86 GHz; microwave power, 10.00 mW; center field, 3454.00 G; modulation amplitude, 0.800 G; modulation frequency, 100.00 kHz; sweep width, 120.0 G; conversion time, 25.00 ms; sweep time, 30 s. Samples (5 mM) and Fe(MGD)_2_ (5 mM) mixed in water containing 5% DMSO were transferred quantitatively to quartz capillaries to record the spectra from 3400 to 3500 G. To compare the two complexes, EPR spectra were recorded in the same illumination distance and illumination time by Hg lamp (100 W) at room temperature. 

### 3.5. Binding of the Complexes with Protein

The measurements for binding of [RuCl(qn)(Lbpy)(NO)]X complexes with HSA were performed using the fluorescence quenching experiments and MALDI-TOF-MS spectra. Fluorescence spectra of HSA (10 μM) in 20 mM PB buffer (pH 7.4) and different concentrations of the complexes (0~80 μM) were measured in the 280 nm excitation wavelength. The emission wavelength was in a range of 290–520 nm. The excitation and emission slit-width were both 5 nm. The experiment was repeated three times. Then, MALDI-TOF-MS spectra of HSA (5 mg/mL) and the complexes (the ratio was 1:8) in 20 mM PB buffer (pH 7.4) were recorded using an ultraflextreme high-resolution mass spectrometer from 30,000 to 150,000 with 2,5-dihydroxybenzoic acid (DHB) as the matrix. 

To observe the influence on the NO release of complexes after interacting with HSA, EPR technology was operated. The NO release situations from the mixture of [RuCl(qn)(Lbpy)(NO)]X complexes (5 mM), Fe(MGD)_2_ (5 mM) and HSA (0.625 mM) in water containing 5% DMSO were monitored by EPR under the same parameters as mentioned above. 

### 3.6. Binding of the Complexes with DNA and Photo-Induced DNA Damage

Fluorescence spectroscopy were utilized to analyze the interaction of CT-DNA with [RuCl(qn)(Lbpy)(NO)]X complexes. CT-DNA solid material was dissolved in distilled water and the stock concentration was calculated by determining the 260 nm absorption using a spectrophotometer. Then, the CT-DNA-EB system was prepared by adding 2.5 μM EB and 170 μM CT-DNA to 10 mM tris buffer (pH 7.4). Finally, the titrations were conducted by the addition of complexes dissolved in DMSO step by step to the CT-DNA-EB solution, until a plateau in the fluorescence intensity was reached (Ru-Lbpy_1_:0~200 μM and Ru-Lbpy_2_:0~500 μM). All the experiments were stirred gently at room temperature for 5 min. The fluorescence spectra were collected in the range of 530–750 nm upon 520 nm excitation with 5 nm/5 nm slit width in a 1-cm quartz. The experiment was repeated three times.

Photo-induced plasmid DNA unwinding experiments of two [RuCl(qn)(Lbpy)(NO)]-X complexes were estimated by agarose gel electrophoresis. Complexes were prepared as 5 mM DMSO stock solutions and diluted to needful concentrations with 10 mM Tris-HCl buffer (pH 7.4). As controls, the supercoiled pBR322 DNA (0.05 μg) and the complexes (0~200 μM) in a total volume of 20 μL buffer were incubated for 30 min in the dark. In order to compare the cleavage action of DNA after illumination, the mixture of pBR322 DNA and the complexes was irradiated for 10 min with a Xe light source. Finally, the mixed solution was electrophoresed at 70 V through 1% agarose gel immersed in 1 × trimethylol aminomethane-boric acid (TBE) buffer for 40 min.

### 3.7. Cellular Culture

The cytotoxicity and photocytotoxicity of two [RuCl(qn)(Lbpy)(NO)]X complexes were evaluated against HeLa cells using CCK8 assay. Hela cells were cultured at 37 °C/5% CO_2_ atmosphere with DMEM medium adding 10% (*v*/*v*) FBS and 1% (*v*/*v*) penicillin/streptomycin. The cells were seeded on a 96-well plate with a density of 5 × 10^4^ cells/mL and incubated for 12 h. Then, the cultured cells were treated with the cell culture medium containing different concentrations of [RuCl(qn)(Lbpy)(NO)]X complexes (0, 1, 5, 10, 50, 500 μM) and further incubated for 24 h. Later, CCK-8 solution (100 μL, 1 mg/mL) diluted with the cell culture medium was added to each well and the plate was further incubated for 3 h. After the incubation, the absorbance was recorded at 450 nm using a microplate reader. As control, another plate was operated using the same procedure as described above except for the LED lighting 30 min after the addition of the drugs for one hour. Each experiment was repeated at least three times. 

### 3.8. Cellular Imaging of NO

HeLa cells were seeded on fluorescent imaging dish with glass bottom with a diameter of 35 mm and were maintained at a 37 °C/5% CO_2_ incubator for 12 h. The cells were washed twice with PBS, treated with NO probe DAX-J2 Red (5.0 μM) and incubated at 37 °C for 30 min. The cells were then treated with [RuCl(qn)(Lbpy)(NO)]X complexes (10 μM) for 20 min, washed twice with PBS, and illuminated 5, 10 min by a LED light with the current of 0.3 A and the wavelength of 420 nm. After each light exposure, it was immediately analyzed under a confocal fluorescence imaging system. Excitation was performed with a laser at λ = 561 nm and the emission wavelength was recorded in the range of 579–701 nm. Cells with only the probe but no complex were used as the control.

## 4. Conclusions

In summary, two light-activated NO donors [RuCl(qn)(Lbpy)(NO)]X based on 8-hydroxy-quinoline and 2,2′-bipyridine derivatives were synthesized and characterized. The crystal structure of [RuCl(qn)(Lbpy_2_)(NO)]NO_3_ complex shows a typical {Ru-NO}^6^ conformation. The NO release ability of complexes is not only related to the substitution ligand, but also external light irradiation, including optical wavelength, density and time. The fluorescence studies and MALDI-TOF-MS spectra support the assertion that the complexes could bind to protein and HSA could be a delivery system for the complexes. According to EPR spectra, the interaction of the complexes with HSA will form an HSA-Ru complex adduct to promote the NO release upon irradiation, which makes a foundation for bio-application in photodynamic therapy. In addition, Ru-Lbpy_1_ has stronger binding ability with DNA than Ru-Lbpy_2_ and illumination promotes the DNA cleavage activity of the complexes. Notably, the hydrogen bond on the Ru-Lbpy_1_ carboxyl group is also in favor of the combination between the complex and HSA/DNA. Moreover, confocal fluorescence imaging shows that photo-induced NO release could be detected in the cellular environment. The results indicate that different substituted ligands in the complexes affect the interaction mode with biomacromolecules and adjust their biological activities, it will guide the rational design of NO donors and metal complex-based drugs.

## Figures and Tables

**Table 1:** Crystal structure parameters of Ru-Lbpy_2_ complex

Chemical formula	C_25_H_24_ClN_5_O_10_Ru	α (°)	105.601(2)
Formula weight	691.01	β (°)	101.476(3)
Temperature (K)	296	γ (°)	107.325(2)
Color	brown	Volume (Å^3^)	1390.9(2)
Size (mm)	0.35 × 0.35 × 0.33	Z	2
Crystal system	Triclinic	Calculated density D_x_ g/cm^−3^	1.650
Space group a (Å) b (Å) c (Å)	*P*-1 10.5852(9) 11.4031(10) 13.1528(12)	R [F^2^ > 2σ(F^2^)] wR_2_ (F^2^) GOOF CCDC number	0.0663 0.1663 1.023 2054814

**Table 2:** Selected bond distances [Å] and angles [°] for Ru-Lbpy_2_ complex.

Ru1—Cl1	2.373(2)	N1—Ru1—N2	79.3(2)
Ru1—N1	2.068(5)	N1—Ru1—N3	166.6(2)
Ru1—N2	2.073(6)	N1—Ru1—N4	95.9(3)
Ru1—N3	2.070(5)	N1—Ru1—O5	85.9(2)
Ru1—N4	1.752(6)	N2—Ru1—N3	95.6 (2)
Ru1—O5	1.980(4)	N2—Ru1—N4	92.4(3)
N4—O6	1.101(9)	N2—Ru1—O5	84.8(2)
Cl1—Ru1—N1	96.0(2)	N3—Ru1—N4	96.7(3)
Cl1—Ru1—N2	172.0(2)	N3—Ru1—O5	81.3(2)
Cl1—Ru1—N3	87.6(2)	N4—Ru1—O5	176.4(2)
Cl1—Ru1—N4	94.4(2)	Ru1—N4—O6	177.6(6)
Cl1—Ru1—O5	88.5(1)		

**Table 3:** The binding constant (K_b_) and number of average binding site (n) of two Ru-Lbpy complexes with HSA and DNA.

	HSA		DNA	
Complex	K_b_/(L·mol^−1^)	n	K_b_/(L·mol^−1^)	n
Ru-bpy_1_	4.83 ± 0.09 × 10^6^	1.46	2.14 ± 0.08 × 10^4^	1.20
Ru-bpy_2_	7.41 ± 0.08 × 10^4^	1.09	5.52 ± 0.16 × 10^3^	1.14

## Data Availability

CCDC 2054814 contains the supplementary crystallographic data for [RuCl(qn)(Lbpy_2_)(NO)]NO_3_. These data can be obtained free of charge via http://www.ccdc.cam.ac.uk/conts/retrieving.html, accessed on 22 April 2021, or from the Cambridge Crystallographic Data Centre, 12 Union Road, Cambridge CB2 1EZ, UK.

## Supplementary Materials

The following are available online. Figure S1: Nuclear magnetic resonance (^1^H NMR) of Ru-Lbpy_1_, Figure S2: ^1^H NMR of Lbpy_2_, Figure S3: ^1^H NMR of Ru-Lbpy_2__,_ Figure S4: Electrospray ionization mass spectrometry (ESI-MS) of Ru-Lbpy_1__,_ Figure S5: ESI-MS of Ru-Lbpy_2__,_ Figure S6: Ultraviolet–visible (UV-vis) spectra of Lbpy_1_ and Ru-Lbpy_1__,_ Figure S7: UV-vis of Lbpy_2_ and Ru-Lbpy_2__,_ Figure S8: Infrared (IR) (KBr pellet) of Ru-Lbpy_1__,_ Figure S9: IR (KBr pellet) of Ru-Lbpy_2__,_ Figure S10: HeLa cells viability treated with Ru-Lbpy_1_ and Ru-Lbpy_2_, Table S1: Crystal atomic coordinates and equivalent isotropic temperature factors of Ru-Lbpy_2_, Table S2: Crystal anisotropic displacement parameters of Ru-Lbpy_2__._

## Sample Availability

Samples of the compounds are not available from the authors.
