# Supplementary material for: Synthesis, Biomacromolecular Interactions, Photodynamic NO Releasing and Cellular Imaging of Two [RuCl(qn)(Lbpy)(NO)]X Complexes
Source: Molecules. 2021 Apr 27;26(9):2545. doi: 10.3390/molecules26092545 (PMC8123785; doi:10.3390/molecules26092545)
Supplement: Supplementary file 1 [file molecules-26-02545-s001.zip › molecules-1169971-supplementary.pdf]

Supporting Information

Synthesis, characterization, biomarcomolecular interactions, photodynamic NO releasing and cellular imaging of two [RuCl(qn)(Lbpy)(NO)]X complexes

Luna Song [1,2], Hehe Bai [1,2], Chenyang Liu [1], Wenjun Gong [1],

Ai Wang [1], Li Wang [2], Yi Zhao [3], Xuan Zhao [4], Hongfei Wang [1,2] *

[1] Key Laboratory of Chemical Biology and Molecular Engineering of the Education Ministry, Institute of Molecular Science, Shanxi University, Taiyuan 030006, China

[2] Institute of Environmental Science, College of Chemistry and Chemical Engineering, Shanxi University, Taiyuan 030006, China

[3] Shanxi Key Laboratory of Pharmaceutical Biotechnology, Taiyuan 030006, China

[4] Department of Chemistry, University of Memphis, Memphis, TN 38152, USA

Table of Contents

Figure S1. ^{1}H NMR of Ru-Lbpy$_1$......3

Figure S2. ^{1}H NMR of Lbpy$_2$......4

Figure S3. ^{1}H NMR of Ru-Lbpy$_2$......5

Figure S4. ESI-MS of Ru-Lbpy$_1$......6

Figure S5. ESI-MS of Ru-Lbpy$_2$......6

Figure S6. UV-vis of Lbpy$_1$ and Ru-Lbpy$_1$ 7

Figure S7. UV-vis of Lbpy$_2$ and Ru-Lbpy$_2$ 7

Figure S8. IR (KBr pellet) of Ru-Lbpy$_1$ 8

Figure S9. IR (KBr pellet) of Ru-Lbpy$_2$ 8

Figure S10. Hela cells viability treated with Ru-Lbpy$_1$ and Ru-Lbpy$_2$ 9

Table S1. Crystal atomic coordinates and equivalent isotropic temperature factors of Ru-Lbpy$_2$......10

Table S2. Crystal anisotropic displacement parameters of Ru-Lbpy$_2$......12

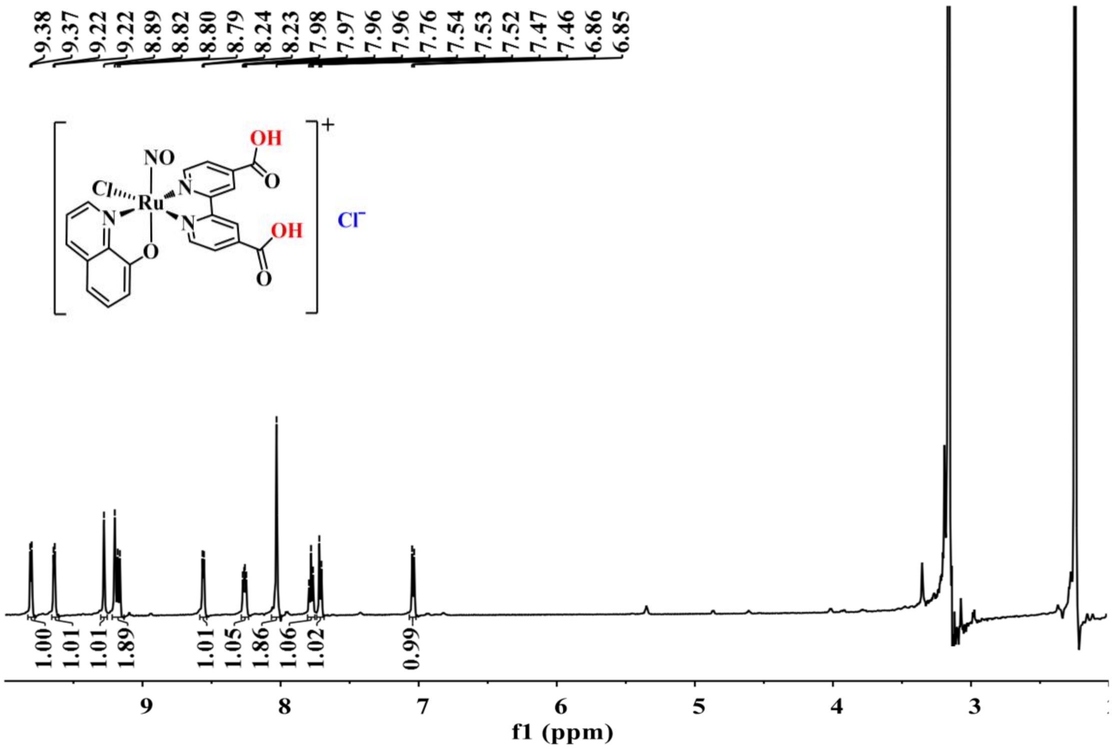

Figure S1. ^{1}H NMR of [RuCl(qn)(Lbpy$_1$)(NO)]Cl (Ru-Lbpy$_1$)

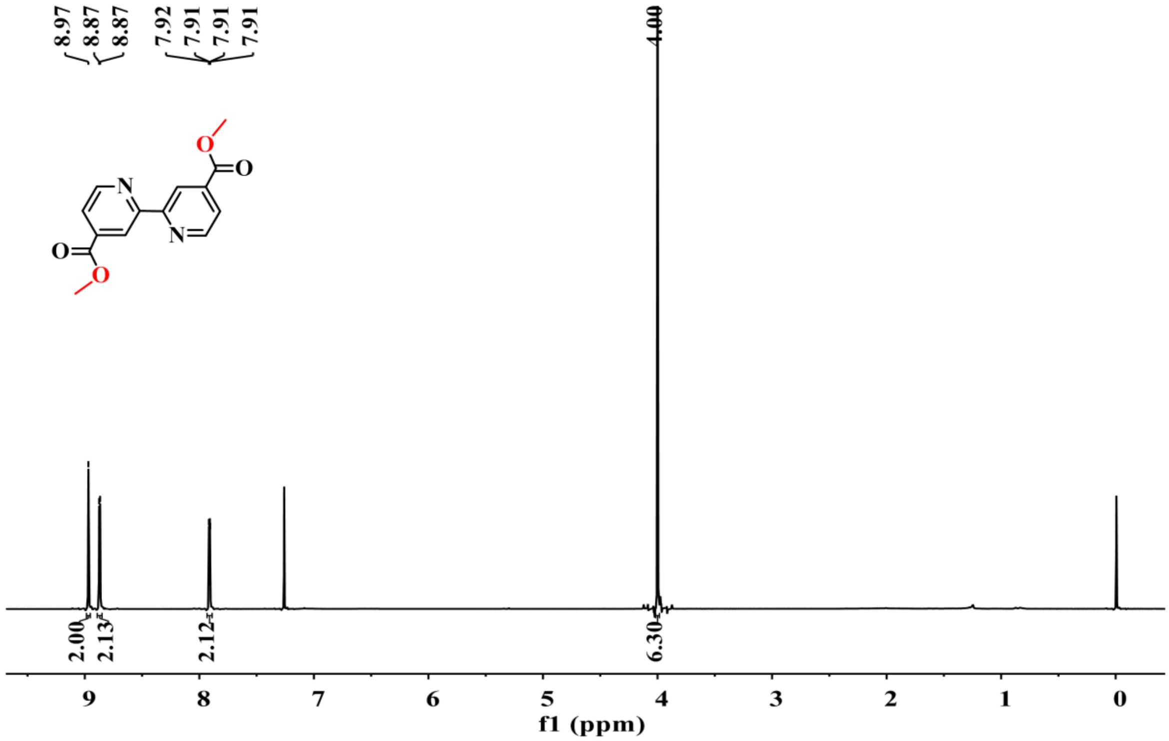

Figure S2. ^{1}H NMR of 4,4′-dimethoxycarbonyl-2,2′-dipyridine (Lbpy$_2$)

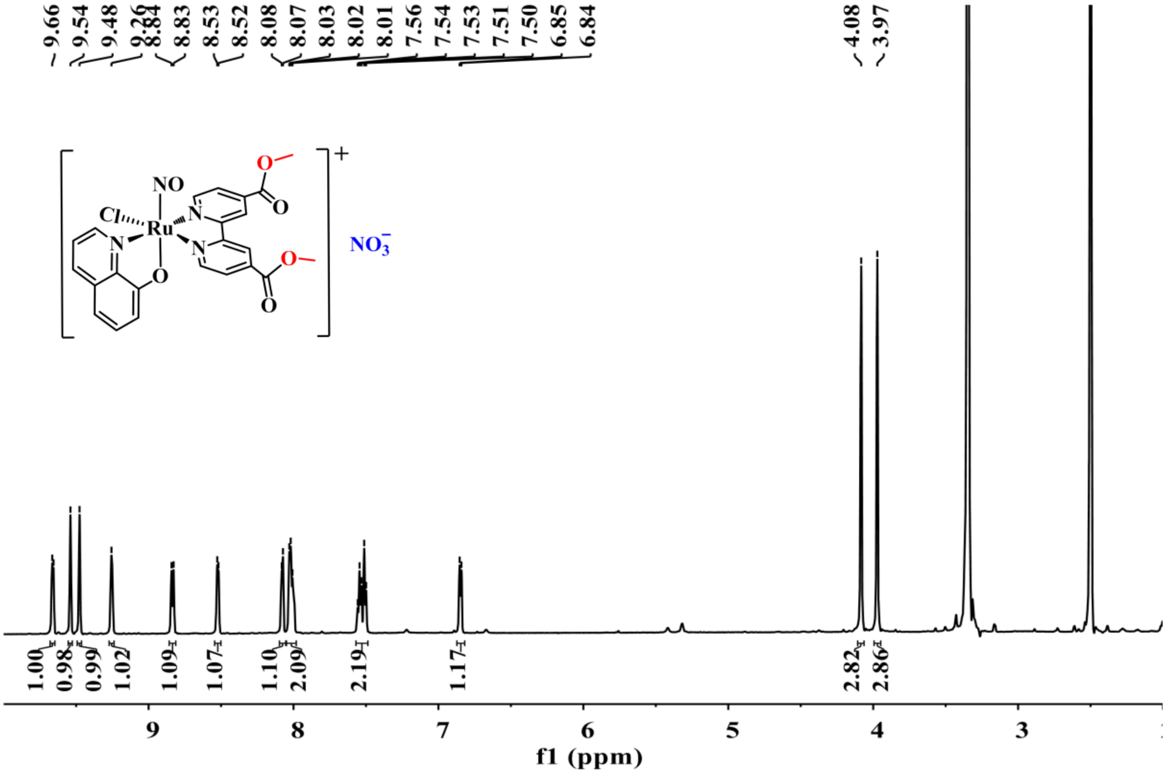

Figure S3. ^{1}H NMR of [RuCl(qn)(Lbpy$_2$)(NO)]NO_3 (Ru-Lbpy$_2$)

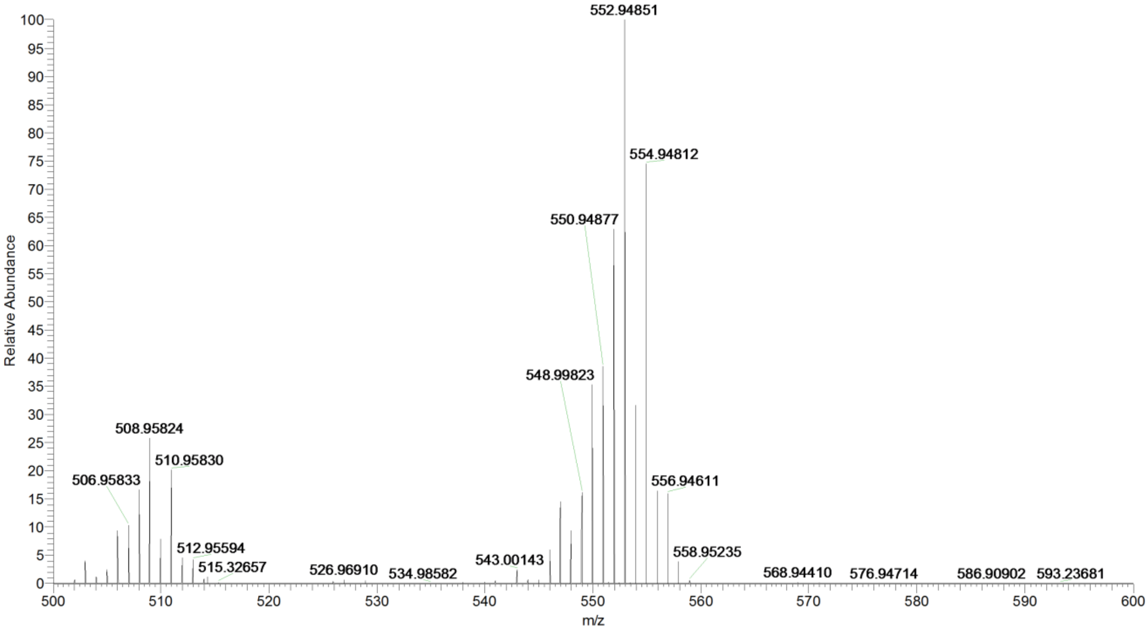

Figure S4. ESI-MS of [RuCl(qn)($Lbpy_1$)(NO)]Cl (Ru-$Lbpy_1$)

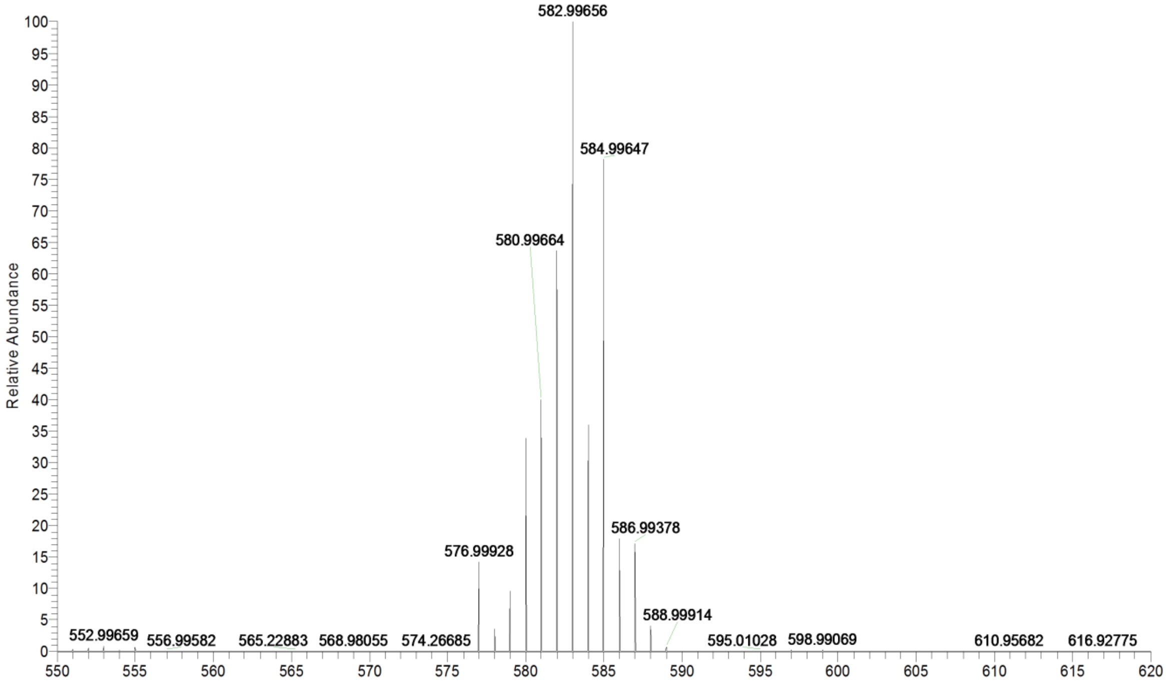

Figure S5. ESI-MS of [RuCl(qn)($Lbpy_2$)(NO)]NO_3 (Ru-$Lbpy_2$)

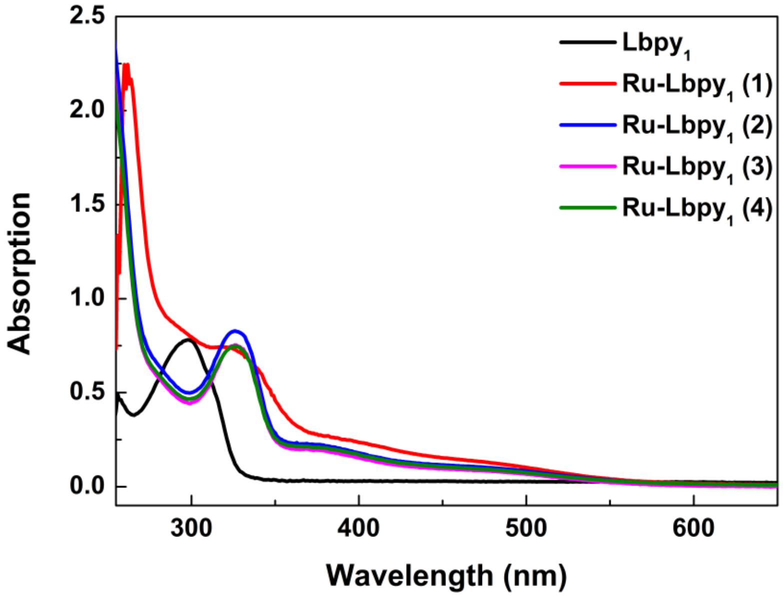

Figure S6. UV-vis of $Lbpy_1$ in DMSO and [RuCl(qn)($Lbpy_1$)(NO)]Cl (Ru-$Lbpy_1$) in (1) DMSO, (2) H_2O, (3) 50 mM PB, pH=7.5 buffer, (4) 50 mM PB, pH=7.5 buffer in a week.

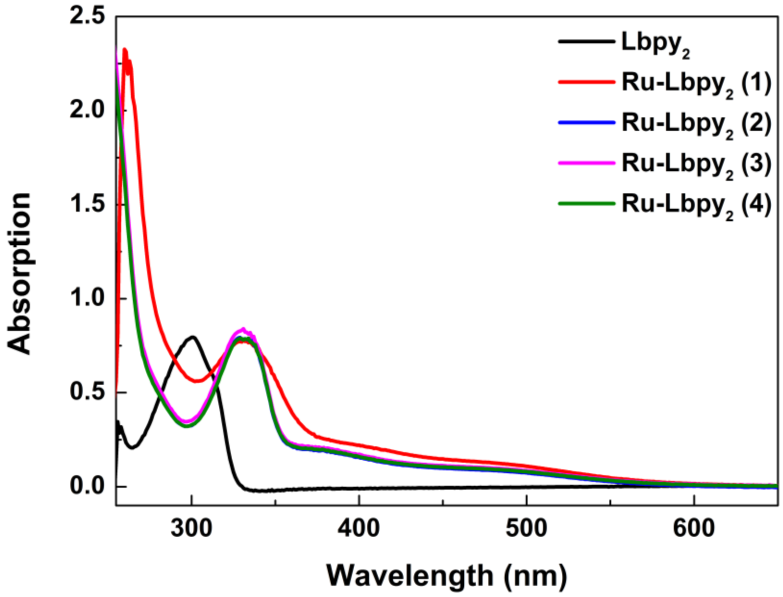

Figure S7. UV-vis of $Lbpy_2$ in DMSO and [RuCl(qn)($Lbpy_2$)(NO)]NO_3 (Ru-$Lbpy_2$) in (1) DMSO, (2) H_2O, (3) 50 mM PB, pH=7.5 buffer, (4) 50 mM PB, pH=7.5 buffer in a week.

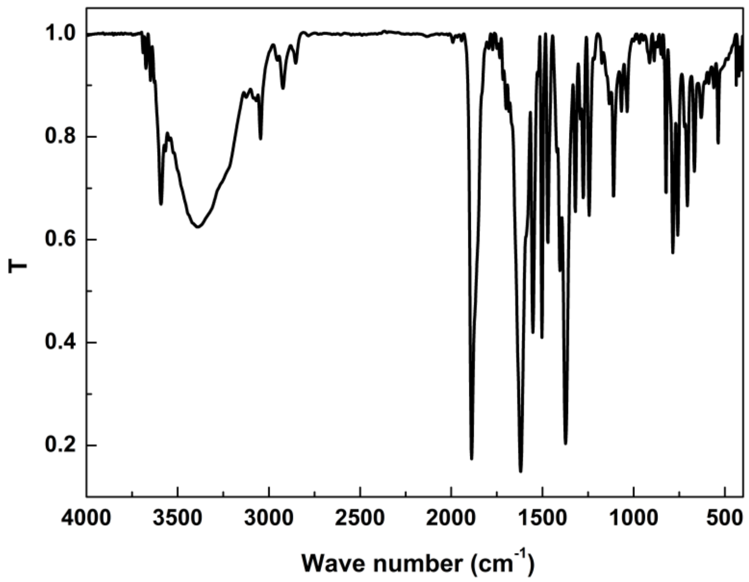

Figure S8. IR of [RuCl(qn)(Lbpy$_1$)(NO)]Cl (Ru-Lbpy$_1$)

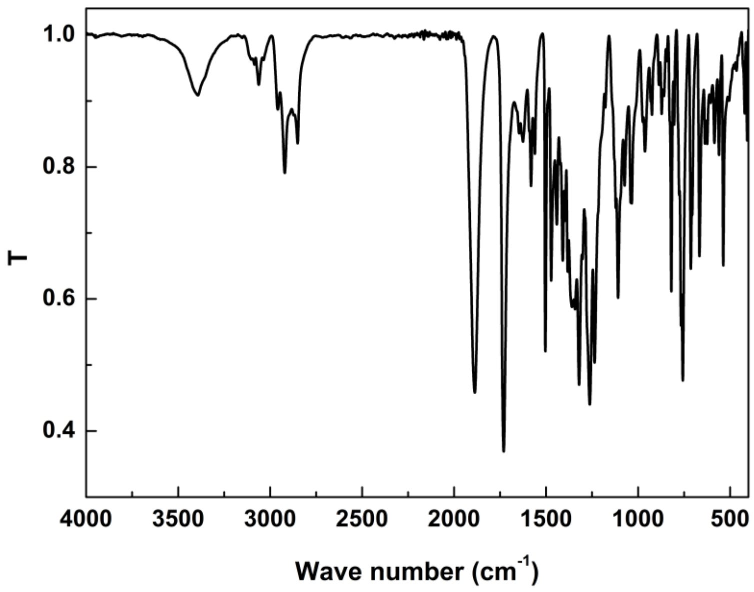

Figure S9. IR of [RuCl(qn)(Lbpy$_2$)(NO)]NO$_3$ (Ru-Lbpy$_2$)

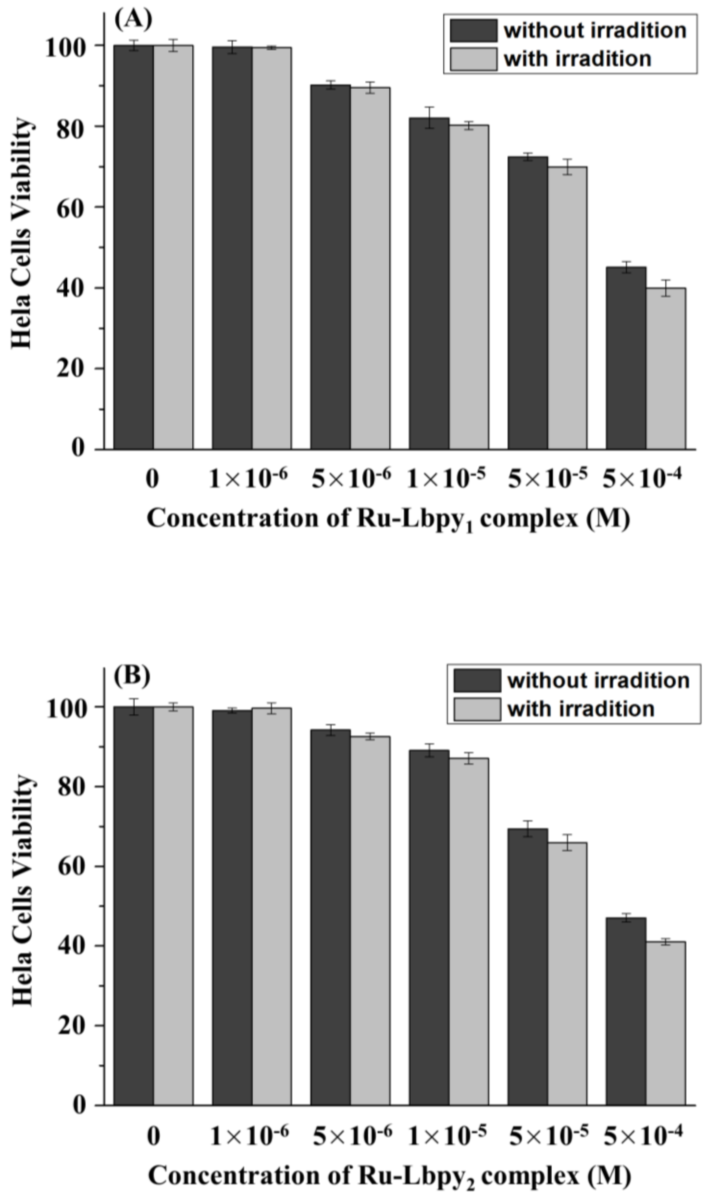

Figure S10. Viability of Hela cells treated with different concentrations of Ru-Lbpy$_1$ (A) and Ru-Lbpy$_2$ (B) complexes for 24 h. The black and gray represent dark condition and light for 30 min, respectively. Each experiment was repeated at least three times.

Table S1. Crystal atomic coordinates and equivalent isotropic temperature factors of Ru-Lbpy$_2$

	x	y	z	U_{iso}
**Ru1**	0.43083(5)	0.31373(5)	0.21151(4)	0.03568(19)
**Cl1**	0.32859(17)	0.35355(18)	0.05295(15)	0.0526(5)
**N1**	0.6344(5)	0.4025(5)	0.2167(4)	0.0375(12)
**N2**	0.5349(5)	0.2674(5)	0.3367(4)	0.0368(12)
**N3**	0.2353(5)	0.1849(5)	0.1922(4)	0.0357(12)
**N4**	0.4237(5)	0.4512(6)	0.3061(5)	0.0476(14)
**N5**	0.2004(10)	0.3313(9)	0.4935(4)	0.088(2)
**O1**	1.0878(5)	0.6091(6)	0.1688(5)	0.0682(16)
**O2**	1.1368(6)	0.5170(7)	0.2889(6)	0.089(2)
**O3**	0.8964(6)	0.1791(6)	0.5658(5)	0.0735(17)
**O4**	0.7132(6)	0.0906(6)	0.6169(5)	0.0704(16)
**O5**	0.4364(4)	0.1524(4)	0.1112(3)	0.0395(10)
**O6**	0.4237(6)	0.5384(6)	0.3677(6)	0.094(2)
**O7**	0.6627(17)	0.8743(17)	0.3709(12)	0.106(7)
**O7'**	0.591(3)	0.766(3)	0.317(2)	0.133(13)
**O8**	0.1047(8)	0.3643(7)	0.4742(6)	0.102(2)
**O9**	0.1833(12)	0.2157(12)	0.4848(9)	0.169(4)
**O10**	0.3177(15)	0.4218(14)	0.5186(10)	0.073(6)
**O11**	0.2592(14)	0.3544(14)	0.5960(12)	0.118(6)
**C1**	0.6762(7)	0.4745(6)	0.1554(5)	0.0439(16)
**C2**	0.8118(7)	0.5234(6)	0.1594(5)	0.0432(16)
**C3**	0.9097(6)	0.4983(6)	0.2280(5)	0.0390(15)
**C4**	0.8673(6)	0.4266(6)	0.2924(5)	0.0375(14)
**C5**	0.7293(6)	0.3779(6)	0.2858(5)	0.0340(14)
**C6**	0.6727(6)	0.3020(6)	0.3520(5)	0.0325(13)
**C7**	0.7528(7)	0.2666(6)	0.4263(5)	0.0398(15)
**C8**	0.6883(7)	0.1971(6)	0.4853(5)	0.0426(16)
**C9**	0.5495(7)	0.1680(7)	0.4719(6)	0.0454(16)
**C10**	0.4747(7)	0.2042(6)	0.3964(5)	0.0420(15)

**C11**	1.0582(7)	0.5414(6))	0.2324(6)	0.0428(16)
**C12**	1.2305(9)	0.6505(10)	0.1653(9)	0.085(3)
**C13**	0.7658(8)	0.1500(7)	0.5640(6)	0.0504(18)
**C14**	0.9767(9)	0.1280(10)	0.6339(8)	0.091(3)
**C15**	0.3221(6)	0.0472(6)	0.0805(5)	0.0384(15)
**C16**	0.3023(8)	-0.0734(7)	0.0103(6)	0.0507(18)
**C17**	0.1768(9)	-0.1786(7)	-0.0162(6)	0.059(2)
**C18**	0.0710(8)	-0.1670(7)	0.0249(6)	0.059(2)
**C19**	0.0864(7)	-0.0445(7)	0.0970(6)	0.0458(17)
**C20**	0.2127(6)	0.0614(6)	0.1240(5)	0.0366(14)
**C21**	0.1382(7)	0.2054(7)	0.2351(6)	0.0464(16)
**C22**	0.0119(8)	0.1034(9)	0.2119(6)	0.057(2)
**C23**	-0.0138(7)	-0.0184(8)	0.1443(6)	-0.0522(19)
**C24**	0.5905(14)	0.8996(14)	0.2023(12)	0.132(5)
**C25**	0.6431(17)	0.8304(19)	0.2562(13)	0.148(6)

Table S2. Crystal anisotropic displacement parameters of Ru-Lbpy$_2$

	U11	U22	U33	U23	U13	U12
**Ru1**	0.0297(3)	0.0334(3)	0.0381(3)	0.0128(2)	0.0005(2)	0.0099(2)
**Cl1**	0.0405(9)	0.0602(11)	0.0528(10)	0.0291(9)	-0.0011(8)	0.0146(8)
**N1**	0.028(3)	0.033(3)	0.044(3)	0.017(2)	0.000(2)	0.005(2)
**N2**	0.034(3)	0.036(3)	0.035(3)	0.011(2)	0.004(2)	0.011(2)
**N3**	0.031(3)	0.038(3)	0.037(3)	0.016(2)	0.005(2)	0.011(2)
**N4**	0.034(3)	0.043(4)	0.053(4)	0.008(3)	-0.003(3)	0.014(3)
**N5**	0.089(3)	0.087(3)	0.089(3)	0.0289(19)	0.0243(19)	0.038(2)
**O1**	0.049(3)	0.093(4)	0.097(4)	0.070(4)	0.033(3)	0.034(3)
**O2**	0.051(3)	0.133(6)	0.127(5)	0.103(5)	0.032(4)	0.038(4)
**O3**	0.055(3)	0.095(4)	0.093(4)	0.072(4)	0.013(3)	0.031(3)
**O4**	0.060(3)	0.086(4)	0.072(4)	0.058(3)	0.012(3)	0.015(3)
**O5**	0.029(2)	0.038(2)	0.046(3)	0.010(2)	0.0099(19)	0.0091(19)
**O6**	0.071(4)	0.073(4)	0.095(5)	-0.022(4)	-0.008(3)	0.036(3)
**O7**	0.111(7)	0.109(8)	0.105(7)	0.040(4)	0.032(4)	0.048(4)
**O7'**	0.132(13)	0.136(13)	0.132(13)	0.050(6)	0.041(5)	0.047(6)
**O8**	0.098(4)	0.115(4)	0.103(4)	0.037(3)	0.031(3)	0.052(3)
**O9**	0.170(4)	0.168(4)	0.171(4)	0.065(2)	0.050(2)	0.062(2)
**O10**	0.070(6)	0.072(6)	0.075(6)	0.024(3)	0.020(3)	0.027(3)
**O11**	0.117(6)	0.117(6)	0.118(6)	0.039(3)	0.033(3)	0.047(3)
**C1**	0.037(4)	0.042(4)	0.047(4)	0.024(3)	-0.003(3)	0.009(3)
**C2**	0.042(4)	0.041(4)	0.044(4)	0.024(3)	0.003(3)	0.011(3)
**C3**	0.040(4)	0.033(3)	0.041(4)	0.015(3)	0.009(3)	0.009(3)
**C4**	0.034(3)	0.037(3)	0.038(3)	0.015(3)	0.015(3)	0.015(3)
**C5**	0.035(3)	0.030(3)	0.031(3)	0.011(3)	0.002(3)	0.009(3)
**C6**	0.032(3)	0.030(3)	0.029(3)	0.009(3)	0.000(2)	0.009(3)
**C7**	0.034(3)	0.040(4)	0.041(4)	0.016(3)	0.004(3)	0.011(3)
**C8**	0.046(4)	0.038(4)	0.039(4)	0.019(3)	0.004(3)	0.010(3)
**C9**	0.040(4)	0.050(4)	0.046(4)	0.026(3)	0.010(3)	0.011(3)

**C10**	0.036(4)	0.046(4)	0.045(4)	0.020(3)	0.009(3)	0.015(3)
**C11**	0.043(4)	0.044(4)	0.051(4)	0.029(3)	0.013(3)	0.019(3)
**C12**	0.062(6)	0.111(8)	0.127(8)	0.084(7)	0.053(6)	0.041(5)
**C13**	0.047(4)	0.046(4)	0.049(4)	0.022(4)	0.000(3)	0.009(3)
**C14**	0.066(6)	0.125(8)	0.109(8)	0.089(7)	0.009(5)	0.041(6)
**C15**	0.033(3)	0.038(4)	0.039(4)	0.016(3)	0.002(3)	0.010(3)
**C16**	0.058(5)	0.041(4)	0.050(4)	0.015(3)	0.009(3)	0.019(3)
**C17**	0.071(5)	0.036(4)	0.052(5)	0.012(3)	0.002(4)	0.011(4)
**C18**	0.054(5)	0.044(5)	0.058(5)	0.024(4)	-0.005(4)	0.001(3)
**C19**	0.040(4)	0.049(4)	0.044(4)	0.028(3)	-0.002(3)	0.009(3)
**C20**	0.033(3)	0.037(4)	0.034(3)	0.016(3)	-0.002(3)	0.009(3)
**C21**	0.048(4)	0.056(4)	0.045(4)	0.025(3)	0.015(3)	0.027(4)
**C22**	0.045(4)	0.090(6)	0.060(5)	0.047(5)	0.025(4)	0.031(4)
**C23**	0.028(4)	0.066(5)	0.058(5)	0.038(4)	0.001(3)	0.004(3)
**C24**	0.128(11)	0.162(13)	0.145(12)	0.095(11)	0.047(9)	0.067(10)
**C25**	0.151(13)	0.214(17)	0.146(14)	0.112(14)	0.062(11)	0.106(13)
